# Reversal of severe angioproliferative pulmonary arterial hypertension and right ventricular hypertrophy by combined phosphodiesterase-5 and endothelin receptor inhibition

**DOI:** 10.1186/s12967-014-0314-y

**Published:** 2014-11-26

**Authors:** Maria A Cavasin, Kimberly M Demos-Davies, Katherine B Schuetze, Weston W Blakeslee, Matthew S Stratton, Rubin M Tuder, Timothy A McKinsey

**Affiliations:** Department of Medicine, Division of Cardiology, University of Colorado Denver, Aurora, CO USA; Department of Medicine, Program in Translational Lung Research, Division of Pulmonary Sciences and Critical Care Medicine, University of Colorado Denver, Aurora, CO USA

**Keywords:** Endothelin, Phosphodiesterase, Pulmonary hypertension, Remodeling

## Abstract

**Background:**

Patients with pulmonary arterial hypertension (PAH) are treated with vasodilators, including endothelin receptor antagonists (ERAs), phosphodiesterase-5 (PDE-5) inhibitors, soluble guanylyl cyclase activators, and prostacyclin. Despite recent advances in pharmacotherapy for individuals with PAH, morbidity and mortality rates in this patient population remain unacceptably high. Here, we tested the hypothesis that combination therapy with two PAH drugs that target distinct biochemical pathways will provide superior efficacy relative to monotherapy in the rat SU5416 plus hypoxia (SU-Hx) model of severe angioproliferative PAH, which closely mimics the human condition.

**Methods:**

Male Sprague Dawley rats were injected with a single dose of SU5416, which is a VEGF receptor antagonist, and exposed to hypobaric hypoxia for three weeks. Rats were subsequently housed at Denver altitude and treated daily with the PDE-5 inhibitor, tadalafil (TAD), the type A endothelin receptor (ET_A_) antagonist, ambrisentan (AMB), or a combination of TAD and AMB for four additional weeks.

**Results:**

Monotherapy with TAD or AMB led to modest reductions in pulmonary arterial pressure (PAP) and right ventricular (RV) hypertrophy. In contrast, echocardiography and invasive hemodynamic measurements revealed that combined TAD/AMB nearly completely reversed pulmonary hemodynamic impairment, RV hypertrophy, and RV functional deficit in SU-Hx rats. Efficacy of TAD/AMB was associated with dramatic reductions in pulmonary vascular remodeling, including suppression of endothelial cell plexiform lesions, which are common in human PAH.

**Conclusions:**

Combined therapy with two vasodilators that are approved for the treatment of human PAH provides unprecedented efficacy in the rat SU-Hx preclinical model of severe, angioproliferative PAH.

## Background

Pulmonary arterial hypertension (PAH) is associated with dramatic structural remodeling of small pulmonary arteries (PAs) [1]. The remodeling process is due to excessive proliferation of fibroblasts, endothelial cells and smooth muscle cells, and correlates with vascular inflammation and adventitial fibrosis. Occlusion of PAs, coupled with aberrant vasoconstriction, causes severe increases in pulmonary vascular resistance (PVR) and often culminates in right-sided heart failure. As such, it has been proposed that anti-proliferative agents should be used in combination with vasodilators for the treatment of PAH. To address this hypothesis, a Phase III clinical trial was performed with the anti-cancer agent imatinib in patients with PAH and receiving background standard-of-care (SOC) therapy [2]; current SOC for PAH typically involves the use of vasodilators, including endothelin receptor antagonists (ERAs), phosphodiesterase-5 (PDE-5) inhibitors, and prostacyclins [3]. Imatinib treatment improved exercise tolerance and pulmonary hemodynamics in PAH patients. However, despite functional improvements, imatinib caused serious adverse side effects, and thus will not be developed further for the PAH indication [2]. As such, there remains a significant unmet medical need with regard to treatment of PAH in humans.

To model PAH pre-clinically, rodents are often exposed to chronic hypoxia, which causes pulmonary vasoconstriction and right ventricular (RV) hypertrophy [4,5]. An alternative rodent model is based on administration of the plant alkyloid, monocrotaline, which is thought to trigger PAH by altering pulmonary artery endothelial cell function [6]. Despite their widespread use and utility, neither model exhibits the obliterative vascular lesions found in human PAH. A breakthrough in PAH research was provided by the discovery that combining hypoxia with the VEGF receptor inhibitor, SU5416, in rats results in progressive and severe PAH characterized by occlusive neointima and complex plexiform lesions reminiscent of those found in lungs of patients with PAH [7,8]. In this model, which will hereafter be referred to as SU-Hx, rats develop RV failure and PAH that is directly correlated with the degree of occluded vessels [9].

SOC therapies for PAH have exhibited only minimal efficacy in the SU-Hx model, which is consistent with the palliative actions of approved PAH drugs, and the inability of these drugs to significantly prolong lifespan in PAH patients. For example, the soluble guanylate cyclase (sGC) agonist riociguat lowered PA pressure (PAP) by approximately 15% in SU-Hx rats, and the PDE-5 inhibitor, sildenafil, was even less effective in the model [10]. The ERA bosentan modestly reduced RV hypertrophy in SU-Hx rats, and prevented further increases in RV systolic pressure (RVSP) when delivered starting on day 10 of a 21-day study [11]. *Ex vivo*, BQ123, a peptide ERA, was able to partially block spontaneous vasoconstriction in blood-perfused lungs excised from SU-Hx rats [9].

We hypothesized that, relative to monotherapy, simultaneous use of two FDA-approved PAH drugs that target distinct but redundant biochemical pathways will provide superior efficacy in the SU-Hx model. To test this hypothesis, the PDE-5 inhibitor, tadalafil (TAD) [12,13], was tested in combination with the type A endothelin receptor (ET_A_) antagonist, ambrisentan (AMB) [14,15], for ability to reverse pre-existing PAH in SU-Hx rats. The data presented here reveal profound and unparalleled efficacy of combined TAD/AMB in the rat SU-Hx model.

## Methods

### Experimental animals

Animal experiments were approved by the Institutional Animal Care and Use Committee at the University of Colorado Denver. Ten week-old male Sprague Dawley rats (Charles River Laboratories) were used for all studies. A single dose of SU5416 (Tocris Bioscience; 20 mg/kg) or vehicle control (50% DMSO and 50% of a solution containing 0.5% carboxymethylcellulose sodium, 0.9% sodium chloride, 0.4% Tween-80, 0.9% benzyl alcohol in deionized water) was administered at day zero. Rats receiving SU5416 and were housed in a hypobaric chamber to simulate an altitude of 18,000 feet above sea level and create a hypoxic environment (10% 0_2_). Normoxic control rats were maintained in chambers simulating sea level (21% 0_2_). After three weeks, all animals were transferred to Denver altitude and treated daily for four weeks by oral gavage with vehicle (0.5% hydroxypropyl-methyl cellulose), tadalafil (10 mg/kg), ambrisentan (10 mg/kg), or a combination of tadalafil and ambrisentan (10 mg/kg of each compound). Tadalafil and ambrisentan were obtained from Sequoia Research Products.

### Hemodynamic analysis

Echocardiographic analyses were performed using a Vevo770 (VisualSonics). Animals were anesthetized using 2% isoflurane and their body temperature was maintained at 37°C. Pulse-wave Doppler of pulmonary outflow was recorded in the parasternal short-axis view at the level of the aortic valve. Baseline measurements were obtained one day prior to placing animals in chambers and serially thereafter. RV hemodynamics were assessed at end-point and after the last ultrasound analysis using a pressure-volume system (Scisense); rats were ventilated with 100% oxygen and 2% isoflurane (Hallowell). Systemic blood pressure was monitored with another pressure catheter inserted in the femoral artery and steady-state hemodynamics were recorded. PAP was measured with the same catheter advanced to the main pulmonary artery; correct placement of the catheter was confirmed by observing a significant rise in diastolic pressure as the catheter moved out of the ventricle. For data from all *in vivo* studies, GraphPad Prism software was used to generate graphs and analyze data. ANOVA with Bonferroni’s post-test (*P* < 0.05) was used to determine statistical differences between groups.

### Tissue procurement and analysis

After end-point hemodynamic measurements were obtained, rats were sacrificed by exsanguination. Heparinazed blood was used to measure blood gases (ABL825 Flex, Radiometer). Lungs were flushed with cold saline and the left lobe was inflated with a mixture of 50:50 cryoprotective embedding medium (OCT) and 30% sucrose, then cut longitudinally and snap-frozen in a block containing OCT. For assessment of pulmonary vascular remodeling, lung sections were stained with hematoxylin and eosin. Stereological assessment of intima (i.e., obliterative) and media (i.e., thickening) was performed using a sampling strategy outlined in Stacher et al. [16], which determined that ~40 histological fields provide ~100 hitting points in intima using a 512 point grid. The structure-specific hitting points were normalized by grid points hitting alveolar septa, which should not change in untreated vs. treated SU-Hx rats, therefore providing a key measure of the reference tissue. This provides the volume density of intima or media in relation to alveolar septa, which is dimensionless. Stereological assessment was performed with coded slides, which were assigned using a random number generator.

Frozen lung sections were fixed with 4% paraformaldehyde for 15 minutes prior to staining for von Willebrand factor (vWF; endothelial cells) and α-smooth muscle actin (αSMA; smooth muscle cells). Antibodies: anti-vWF (Abcam, ab6994), Cy3-anti-αSMA (Sigma, C6198), and FITC-anti-rabbit IgG (Invitrogen, A11034). Vectashield mounting medium with DAPI (Vector, H-1500) was used to mount coverslip to slides.

RV was dissected from LV by cutting along the septum and the outer wall of the LV and weighed. Fifty milligram biopsies of RV free wall were flash-frozen in liquid nitrogen for biochemical analysis. Protein lysates were prepared in PBS (pH 7.4) containing 0.5% Triton X-100, 300 mM NaCl and protease/phosphatase inhibitor cocktail (Thermo Fisher) using a Bullet Blender homogenizer (Next Advance). Proteins were resolved by SDS-PAGE, transferred to nitrocellulose membranes (BioRad) and probed with antibodies for RCAN-1 [17] or calnexin (Santa Cruz Biotechnology, sc-11397). Proteins were detected using a SuperSignal West Pico chemiluminescence system (Thermo Scientific) and a FluorChem HD2 imager (Alpha Innotech).

## Results and discussion

### Monotherapy to inhibit PDE-5 or ET_A_ signaling modestly reduces PAH and RV hypertrophy in SU-Hx rats

**Figure 1:**
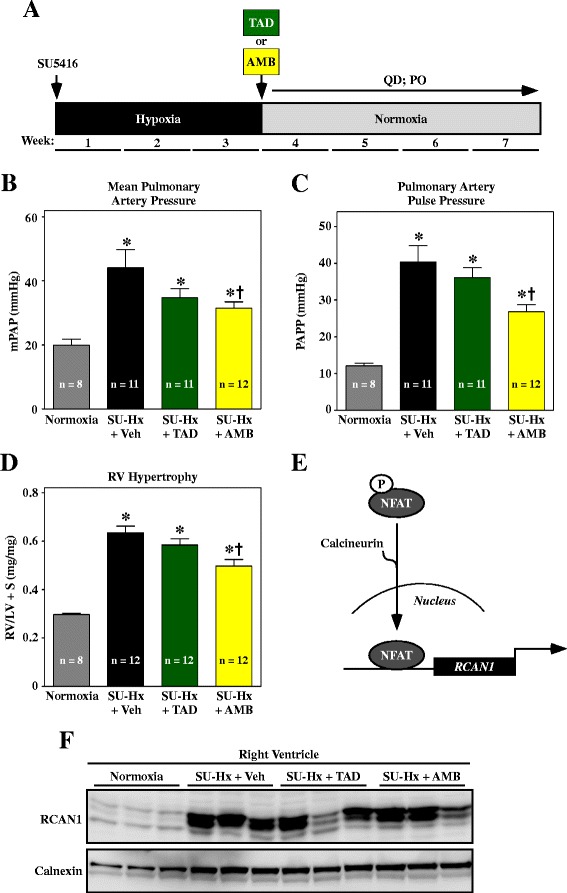
**Monotherapy to block PDE-5 or ET**_**A**_ **signaling modestly reduces pulmonary pressure and RV hypertrophy. (A)** Study design. Animals received 10 mg/kg tadalafil (TAD) or ambrisentan (AMB) once daily by oral gavage starting after week three. **(B–C)** Mean pulmonary arterial pressure (mPAP) and PA pulse pressure (PAPP) were measured invasively at study endpoint. **(D)** RV hypertrophy was assessed by weighing ventricular chambers at the time of necropsy, and is expressed as a ratio to LV + septum (S). **(E)** The gene encoding regulator of calcineurin-1 (RCAN1) is regulated by the nuclear factor of activated T cells (NFAT) transcription factor, which translocates to the nucleus in response to dephosphorylation by the pro-hypertrophic phosphatase, calcineurin. **(F)** RCAN1 expression in RV homogenates was detected by immunoblotting. Calnexin served as a loading control. Values represent mean +/−SEM. *P < 0.05 vs. normoxia; ^†^ *P* < 0.05 vs. SU-Hx + vehicle (Veh). AMB significantly reduced pulmonary arterial pressure and RV hypertrophy, while TAD was without effect. Neither compound consistently blocked RV calcineurin signaling.

Adult male Sprague Dawley (SD) rats were given a single injection of SU5416 and subsequently housed in a hypobaric chamber to simulate an altitude of 18,000 feet above sea level and create a hypoxic environment (10% 0_2_) (Figure 1A). Normoxic control rats were maintained in chambers simulating sea level (21% 0_2_). After three weeks, all animals were transferred to Denver altitude and treated daily (QD) by oral gavage (PO) with TAD, AMB or vehicle control for an additional four weeks.

Invasive hemodynamic measurements were obtained at study endpoint. At the doses employed (10 mg/kg for both compounds), only AMB reduced mean PAP, albeit incompletely (Figure 1B). AMB also lowered PA pulse pressure (PAPP), suggesting that the compound increased arterial compliance in the lungs of SU-Hx rats (Figure 1C). Consistent with the hemodynamic measurements, RV hypertrophy was modestly suppressed by AMB monotherapy, but was unaffected by TAD treatment (Figure 1D).

Calcineurin stimulates cardiac hypertrophy by dephosphorylating the pro-hypertrophic transcription factor, nuclear factor of activated T cells (NFAT). Upon dephosphorylation, NFAT translocates to the nucleus where it drives expression of genes that govern the hypertrophic response. In the heart, calcineurin activity can be monitored indirectly by assessing expression of RCAN1 (regulator of calcineurin 1), since the *RCAN1* gene promoter harbors 15 NFAT binding sites (Figure 1E) [18]. As shown in Figure 1F, calcineurin activity (as measured by RCAN1 expression) was dramatically elevated in RVs of SU-Hx rats and was not significantly altered by TAD or AMB treatment, which is consistent with the minimal effects of these compounds on RV hypertrophy (Figure 1D).

### Combined PDE-5 and ET_A_ inhibition reverses pulmonary hemodynamic impairment and RV hypertrophy in SU-Hx rats

**Figure 2:**
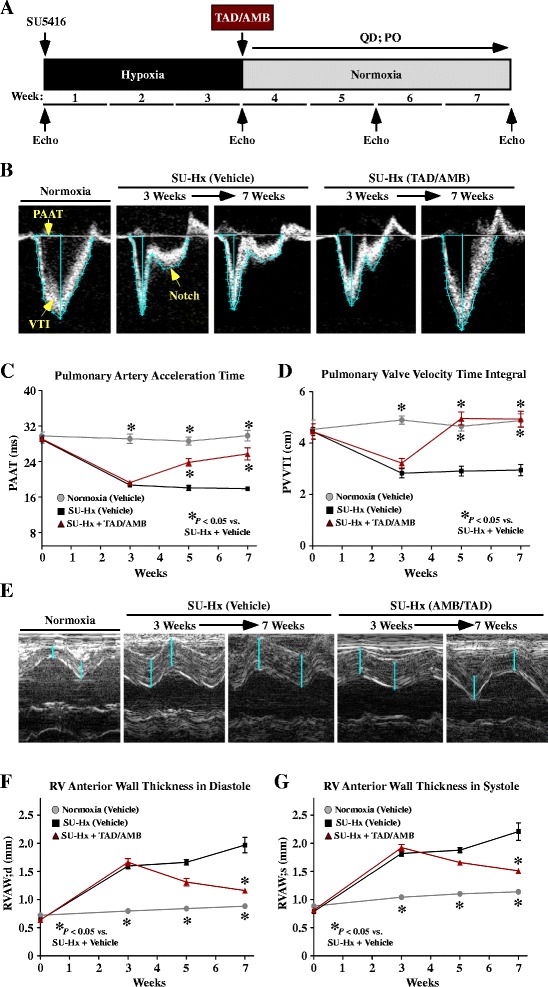
**Combined PDE-5 and ET**_**A**_ **inhibition reverses pulmonary hemodynamic impairment and RV hypertrophy in SU-Hx rats. (A)** Study design. Animals received 10 mg/kg each of tadalafil (TAD) and ambrisentan (AMB) once daily by oral gavage starting after week three. **(B)** Pulmonary artery acceleration time (PAAT) and velocity time integral (VTI) were quantified using Doppler images. Systolic notching of PA blood flow in an SU-Hx rat treated with vehicle is indicated. **(C and D)** PAAT and VTI were significantly reduced in SU-Hx rats compared to normoxic controls, indicating increased pulmonary arterial pressure. PAAT and VTI were rescued by TAD/AMB treatment. **(E–G)** M-mode echocardiographic images revealed increased RV anterior wall thickness in SU-Hx rats, which was dramatically reduced by TAD/AMB treatment. For all graphs, values represent mean +/−SEM. *P < 0.05 vs. SU-Hx + vehicle (Veh). Normoxia (n = 8); SU-Hx (n = 10); SU-Hx + AMB/TAD (n = 12).

We next sought to determine whether simultaneously targeting PDE-5 and ET_A_ would provide superior efficacy over monotherapy with either compound. For these studies, the ability of combined TAD/AMB to reverse pre-existing PAH and RV hypertrophy was assessed. Baseline echocardiographic measurements were obtained prior to exposing male SD rats to SU5416 and three weeks of hypoxia, as described above. As indicated in Figure 2A, serial echocardiography was performed to assess disease progression and effects of dual PDE-5/ET_A_ inhibition.

At three weeks, immediately prior to TAD/AMB treatment, SU-Hx rats exhibited reduced pulmonary artery acceleration time (PAAT) and velocity time integral (VTI). Remarkably, both parameters of PA blood flow were dramatically reversed by TAD/AMB treatment (Figure 2B–D). Reduced pulmonary vascular compliance often causes transient cessation of forward PA blood flow during systole, which is detected by Doppler as a “notch” in the signal. Systolic notching was readily detected in SU-Hx rats, but was absent in animals treated with TAD/AMB (Figure 2B). Consistent with the apparent reduction in PAP in TAD/AMB-treated animals, analysis of M-mode echocardiographic images of the heart revealed a significant regression in RV wall thickness upon dual PDE-5/ET_A_ inhibition (Figure 2E–G).

### Combined PDE-5 and ET_A_ inhibition suppresses PAH and improves RV function in SU-Hx rats

**Figure 3:**
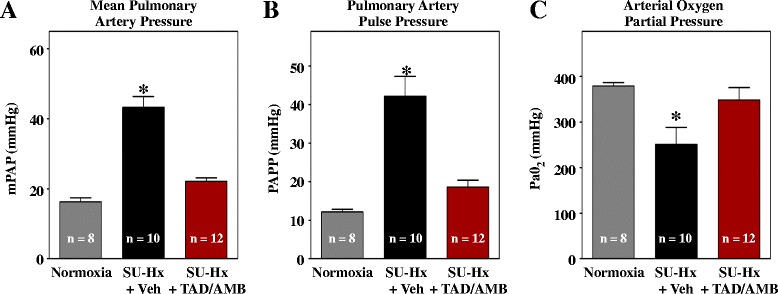
**Combined PDE-5 and ET**_**A**_ **inhibition suppresses pulmonary hypertension in SU-Hx rats. (A–B)** Mean pulmonary arterial pressure (mPAP) and PA pulse pressure (PAPP) were measured invasively at study endpoint. **(C)** The partial pressure of oxygen in arterial blood was significantly increased by TAD/AMB. Values in all groups are higher than normal due to ventilation of animals with 100% O_2_ during pressure-volume analyses.

Invasive hemodynamic measurements obtained at study endpoint revealed that combined TAD/AMB treatment completely normalized PAP (Figure 3A and B). Reduced PAP was associated with increased arterial oxygen partial pressure (Figure 3C), which is likely due to enhanced efficiency of gas exchange between alveoli and arterioles as a consequence of vasodilation.

**Figure 4:**
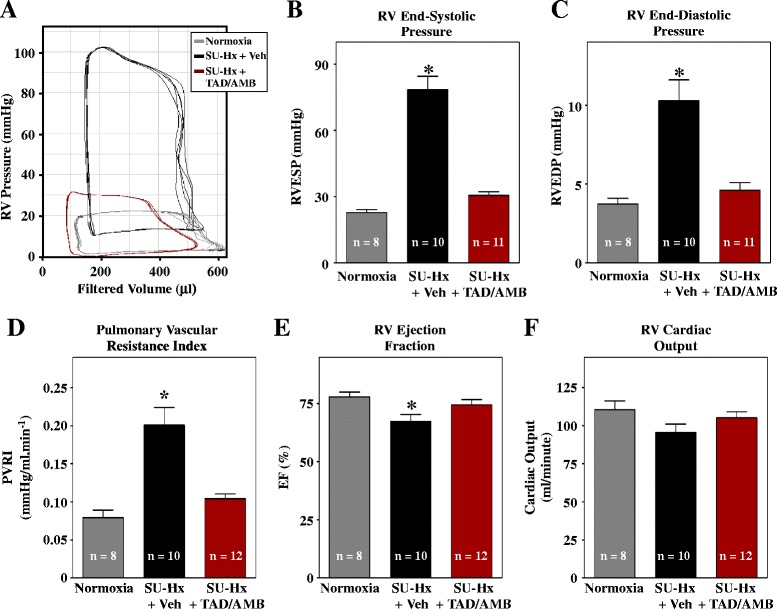
**Combined PDE-5 and ET**_**A**_ **inhibition improves RV function in SU-Hx rats. (A)** Invasive pressure-volume analyses were performed at study endpoint. Representative RV pressure-volume loops are shown. The increase in RV end-systolic **(B)** and end-diastolic pressure **(C)** in SU-Hx rats was completely blocked by combined TAD/AMB treatment. **(D)** Pulmonary vascular resistance, calculated based on cardiac output, was lowered by TAD/AMB. **(E)** RV systolic function, based on ejection fraction (EF), was normalized by TAD/AMB. **(F)** RV cardiac output trended lower in SU-Hx rats, although the decrease was not statistically significant. *P < 0.05 vs. normoxia.

Pressure-volume analyses indicated highly elevated end-systolic and diastolic pressures in RVs of SU-Hx rats, which were reversed by TAD/AMB treatment (Figure 4A–C). Pressure-volume data also demonstrated that TAD/AMB reduced pulmonary vascular resistance (PVR) and improved RV ejection fraction (Figure 4D and E). RV cardiac output trended lower in SU-Hx rats, although the decrease was not statistically significant (Figure 4F).

**Figure 5:**
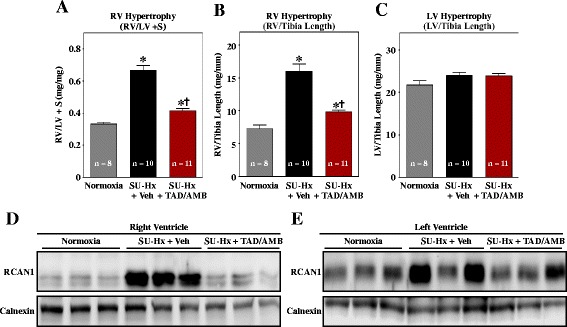
**Combined PDE-5 and ET**_**A**_ **inhibition blocks RV hypertrophy and pro-hypertrophic calcineurin signaling.** RV hypertrophy, as determined by comparing weights of RV vs. LV + septum (S) **(A)** or RV mass to tibia length ratio **(B)** was significantly reduced by combined TAD/AMB treatment. **(C)** LV hypertrophy was not observed in the model. *P < 0.05 vs. normoxia; †*P* < 0.05 vs. SU-Hx + vehicle **(D)** Immunoblotting revealed normalization of RCAN1 expression in RVs of animals treated with TAD/AMB, indicating suppression of calcineurin signaling. Calnexin served as a loading control. **(E)** Elevated RCAN1 expression was observed in LVs of some SU-Hx rats.

In agreement with ultrasound data, RV hypertrophy in SU-Hx rats was dramatically reduced by TAD/AMB treatment (Figure 5A and B); LV mass was not altered in SU-Hx rats (Figure 5C). Suppression of hypertrophy was associated with reduced pro-hypertrophic calcineurin signaling in the RV, as evidenced by inhibition of expression of RCAN1 (Figure 5D). Interestingly, despite that absence of LV hypertrophy, RCAN1 expression was elevated in the LVs of some SU-Hx rats (Figure 5E).

### Combined PDE-5 and ET_A_ inhibition blocks pathological pulmonary vascular remodeling in SU-Hx rats

**Figure 6:**
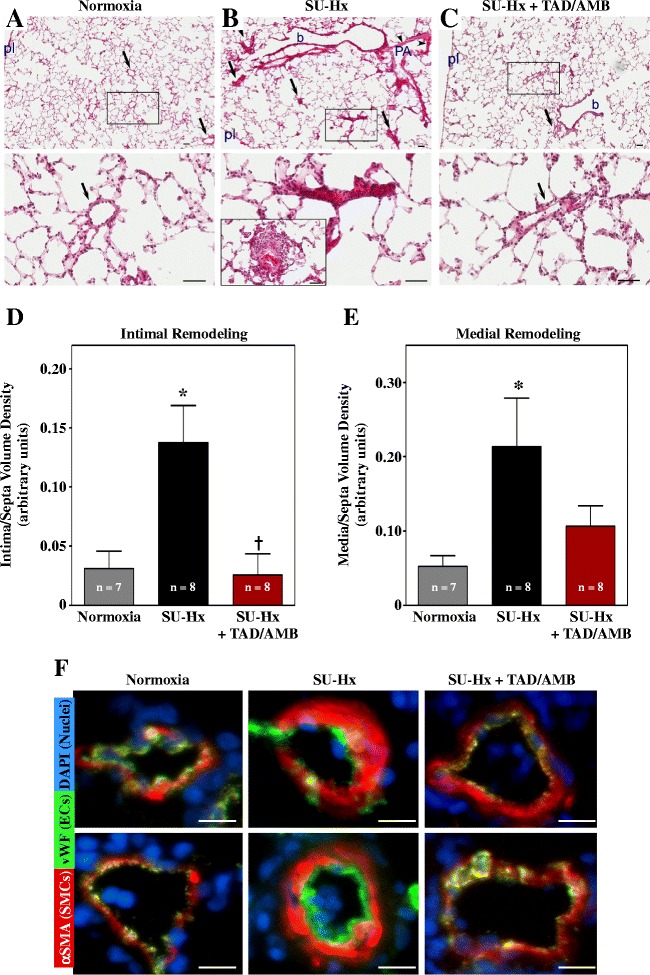
**Combined PDE-5 and ET**_**A**_ **inhibition blocks pathological pulmonary vascular remodeling in SU-Hx rats.** Images of a normoxic control lung **(A)**, severely hypertensive lung in SU-Hx rats **(B)**, and a lung from an SU-Hx animal treated with TAD/AMB for four weeks **(C)**. Note that the SU-Hx lungs have marked muscularization of a pulmonary artery (PA, arrowheads), alongside a bronchiole (b), with several occlusive lesions located within the alveolar unit (arrows). TAD/AMB treatment led to normalization of pulmonary arteries, both at the bronchiolar level (b) as well as in intra-alveolar compartment (arrows), similar to the pulmonary artery morphology (arrows) seen in control lungs. High magnification images of the boxed areas are shown in the lower panels. In addition, in **(B)** the lower panel shows a classical plexiform-like lesion in a vehicle treated SU-Hx rat. This image was taken from a different slide. pl, visceral pleura; magnification bar =50 μm. Intimal occlusion **(D)** and medial thickening **(E)** in SU-Hx rats was reduced by TAD/AMB treatment. Values represent mean +/−SEM. *P < 0.05 vs. normoxia. †*P* < 0.05 vs. SU-Hx + vehicle. **(F)** Representative images of lung arterioles stained for von Willebrand factor (vWF; endothelial cell marker) and alpha smooth muscle actin (αSMA; smooth muscle cell marker). Images are overlaid with DAPI-stained nuclei; magnification bar =10 μm. Vessels from SU-Hx rats exhibited expansion of smooth muscle and endothelial cells, which was reduced by TAD/AMB treatment.

A hallmark of the rat SU-Hx model is severe pulmonary vascular remodeling. Compared to normoxic controls, pulmonary arteries of SU-Hx rats were muscularized and often severely occluded (Figure 6A and B). Classical plexiform lesions were also observed in SU-Hx lungs (Figure 6B). Notably, arterial remodeling was dramatically reduced in TAD/AMB-treated rats (Figure 6C–E), suggesting that efficacy of this drug combination is due, in part, to normalization of the structure of the pulmonary vascular bed. Consistent with this, subsequent antibody staining of lung sections revealed muscularization and endothelial cell expansion in pulmonary arterioles in SU-Hx rats, and this vascular remodeling was reduced by TAD/AMB treatment (Figure 6F).

## Conclusions

Morbidity and mortality rates for individuals with PAH remain unacceptably high, underscoring the need for additional therapeutic options [19]. In the current study, monotherapy with 10 mg/kg tadalafil failed to reduce PAP or RV hypertrophy in the rat SU5416 plus hypoxia model of PH, while the same dose of ambrisentan was mildly efficacious. In sharp contrast, combined treatment with tadalafil and ambrisentan (10 mg/kg each), starting when SU-Hx rats had pre-existing PH and RV hypertrophy, dramatically reversed multiple disease endpoints in the lung and right side of the heart. The findings underscore the promise of combinatorial therapy for PAH based on simultaneous targeting of redundant signaling effectors, PDE-5 and ET_A_, which serve crucial roles in the pathogenesis of PAH.

AMBITION is a Phase III clinical trial designed to assess whether treatment with a combination of TAD and AMB provides superior efficacy over monotherapy in newly diagnosed patients with PAH [20]; preliminary findings from the study were recently announced at the European Respiratory Society meeting in Munich, Germany. Participants in the trial include patients with idiopathic pulmonary arterial hypertension (i.e., PAH or World Health Organization classified Group I PH), as well as individuals with PH due to insults such as structural lung disease, toxin exposure and HIV infection. Based on data presented here, it will be particularly enlightening to determine whether TAD/AMB is efficacious in severe, angioobliterative disease. Additionally, a comparison of AMBITION data with our results will provide an unprecedented opportunity to address the predictive value of the SU-Hx rat model with regard to translating results to the clinical setting. Such comparisons could have a profound impact on the success rate of future drug discovery efforts with experimental compounds for PAH, such as inhibitors of leukotriene B4 biosynthesis [21].

## Abbreviations

PAH: Pulmonary arterial hypertension
PDE-5: Phosphodiesterase-5
AMB: Ambrisentan
TAD: Tadalafil
